# Identification of RD5-Encoded *Mycobacterium tuberculosis* Proteins As B-Cell Antigens Used for Serodiagnosis of Tuberculosis

**DOI:** 10.1155/2012/738043

**Published:** 2012-06-04

**Authors:** Miao-Miao Zhang, Jun-Wei Zhao, Zhan-Qiang Sun, Jun Liu, Xiao-Kui Guo, Wen-Di Liu, Shu-Lin Zhang

**Affiliations:** ^1^Department of Microbiology and Parasitology, Shanghai Jiao Tong University School of Medicine, Shanghai 200025, China; ^2^Department of Immunology, Xinxiang Medical University, Xinxiang 453000, China; ^3^Shanghai Kaidi Biotech Co., Ltd., Shanghai 200433, China; ^4^Wuxi No. 5 People's Hospital, Wuxi 214005, China; ^5^Department of Biochemistry, Henan University of Traditional Chinese Medicine, Zhengzhou 450008, China

## Abstract

Comparative genomic studies have identified several *Mycobacterium tuberculosis*-specific genomic regions of difference (RDs) which are absent in the vaccine strains of *Mycobacterium bovis* BCG and which may be useful in the specific diagnosis of tuberculosis (TB). In this study, all encoded proteins from DNA segment RD5 of *Mycobacterium tuberculosis*, that is, Rv3117–Rv3121, were recombined and evaluated by enzyme-linked immunosorbent assays for antibody reactivity with sera from HIV-negative pulmonary TB patients (*n* = 60) and healthy controls (*n* = 32). The results identified two immunodominant antigens, that is, Rv3117 and Rv3120, both of which revealed a statistically significant antigenic distinction between healthy controls and TB patients (*P* < 0.05). In comparison with the well-known early-secreted antigen target 6 kDa (ESAT-6) (sensitivity 21.7%, specificity 90.6%), the higher detection sensitivity and higher specificity were achieved (Rv3117: sensitivity 25%, specificity 96.9%; Rv3120: sensitivity 31.7%, specificity 96.9%). Thus, the results highlight the immunosensitive and immunospecific nature of Rv3117 and Rv3120 and indicate promise for their use in the serodiagnosis of TB.

## 1. Introduction

Tuberculosis (TB) is an infectious disease caused by *Mycobacterium tuberculosis *(*M. tuberculosis*) and is still a serious global health care problem. In 2010, there were 8.8 million incident cases of TB with 1.45 million deaths [1]. Over the past two decades, drug-resistant TB [2] and TB/HIV coinfection [3] further threatened to undermine tuberculosis control. Until now, there is no simple, rapid, sensitive, and specific test that can be used for diagnosis of tuberculosis. The gold standard for TB diagnosis is the demonstration of *M. tuberculosis* in various body fluids. However, this is often impossible due to the paucibacillary nature of the illness in some cases, especially in smear-negative TB patients. Currently, diagnosis of TB relies mainly on clinical examination and radiographical findings and is further confirmed by bacterial culture and acid-fast bacilli (AFB) smear microscopy [4]. However, it requires long time, and it is also not very sensitive; sometimes other *mycobacteria* found in sputum [5] may decrease the specificity. In recent years several rapid diagnostic techniques have been investigated to determine their ability to improve the diagnosis of TB, such as polymerase chain reaction (PCR) and other methods for amplifying DNA and RNA, though they permit the diagnosis of tuberculosis in as little as several hours, their applicability is limited by low sensitivity, high level of training required, and high cost [6, 7].

In search for rapid and cost-effective diagnostic methods for TB, immunodiagnosis is considered an attractive option, which uses the specific humoral and cellular immune responses of the host to infer the presence of infection or disease. Recently, the antigen-specific *ex vivo* induction of interferon gamma (IFN-*γ*) had been used to detect infection with *M. tuberculosis*. However, the IFN-*γ* release assay could not differentiate the latent tuberculosis infection and active tuberculosis efficiently and cannot be recommended for the diagnosis of tuberculosis in developing countries, as large proportions of the populations in such countries are likely to harbor latent infection with* M. tuberculosis* [8–10]. Historically speaking, serology for the diagnosis of TB has been explored since 1898, when crude cell preparations containing carbohydrates, lipids, and proteins from *M. tuberculosis* or *M. bovis *BCG were used as antigen preparations showing high sensitivity but low specificity [11]. Modern developments in the purification of antigens, generation of monoclonal antibodies and chromatographic techniques, have led to a considerable improvement in specificity [12–18]. Serology has additional advantages in situations when the patient is unable to produce adequate sputum or sputum smear results are negative and TB is extrapulmonary.

Comparative genomic studies have identified several *M. tuberculosis*-specific genomic regions of difference (RDs) which are absent in the avirulent *M. bovis* Bacille Calmette-Guérin (BCG) strains [19, 20] and which may be useful in the specific diagnosis of TB. For example, ESAT-6, culture filtrate protein 10 kDa (CFP-10) [16, 17], Rv3872 [21], and Rv3873 [22] from RD1 were identified as promising diagnostic antigens. Previous studies had also described a protein antigen Rv3425, which was encoded by an open reading frame (ORF) found in RD11 of *M. tuberculosis* and had a strong immunogenicity, suggesting it was a potential candidate for the serodiagnosis of active TB [12, 23]. In this study, we cloned, expressed, and purified the RD5-encoded *M. tuberculosis* recombinant proteins and evaluated the immunoreactivity of the target proteins with sera from HIV-negative pulmonary TB patients and healthy controls, respectively. We aimed at revealing other serological antigens to improve serodiagnostic sensitivity for TB.

## 2. Materials and Methods

### 2.1. Genomic DNA Extraction. *M. tuberculosis*

H_37_Rv was cultured on the Lowenstein-Jensen medium at 37°C for four weeks, and the bacteria (2 mg) were heat-killed at 95°C for 1 h and then treated with 2 mg lysozyme in 1 mL TE buffer at 37°C for 2 h. Subsequently, the cells were subjected to 1% SDS and 50 *μ*g/mL protease K treatments at 55°C for 2 h. The contained DNA was extracted with phenol and chloroform and precipitated with ethanol and 3 mol/L sodium acetate (pH 5.2), followed by being dissolved in TE buffer and stored at −20°C.

### 2.2. Generation of Recombined RD5-Encoded *M. tuberculosis* Proteins

The ORFs corresponding to Rv3117, Rv3118, Rv3119, Rv3120, and Rv3121 were amplified by PCR from the genomic DNA of H_37_Rv, respectively. The primers, restriction endonucleases used, vectors, and annealing temperature for thermal cycle amplification are shown in Table 1. The PCR products were cloned into N-terminal or C-terminal His-tagged expression vector pET-21a, pET-32a, or pET-28b (Novagen, CA, USA) at the restriction sites indicated, and the generated recombinant plasmids were transformed into* E. coli *BL21 (DE3) pLysS for expression. After DNA sequencing, individual transformant was cultured overnight in LB medium containing 50 *μ*g/mL kanamycin or ampicillin, and then the target proteins were induced with 1–50 mmol/L isopropyl-b-D-thiogalactopyranoside (IPTG) for 3 h. The recombinant proteins were purified by immobilized metal affinity chromatography (IMAC) according to the manufacturer's protocol (Bio-Rad Laboratories Ltd., Shanghai, China). Finally, the recombinant protein purity and content were assessed by sodium dodecyl sulfate-polyacrylamide gel electrophoresis (SDS-PAGE) analysis and Bradford's assay using bovine serum albumin (BSA) as the protein standard [24], respectively.

### 2.3. Serum Samples

A total of 60 serum samples (*n* = 60) from HIV-seronegative active TB patients (age range, 1–81 years) and 32 serum samples (*n* = 32) from healthy control subjects (age range, 20–63 years) were collected from the Wuxi No. 5 people's hospital, Jiangsu, China. Active TB patients were diagnosed as previously [25] and were further classified into two groups: (i) smear-positive for acid-fast bacilli (AFB) and culture-positive pulmonary TB (*n* = 48) and (ii) smear-negative culture-positive pulmonary TB (*n* = 12). All of health controls had not previously suffered from TB and had negative chest X-rays and negative sputum culture results for *M. tuberculosis*. Twelve of these were PPD positive at the time the serum samples were taken. All patients had not yet been started with antituberculous chemotherapy when the serum samples were taken. Sera were stored at −20°C.

### 2.4. Enzyme-Linked Immunosorbent Assays (ELISAs)

ELISAs were performed to check the B-cell immune response in humans to RD5-encoded *M. tuberculosis* recombinant proteins and to the well-known antigen ESAT-6 as our previous studies [26, 27]. In brief, 96-well polystyrene flat-bottomed microtiter plates (Costar, USA) were coated with 2–16 *μ*g/mL of either RD5-encoded recombinant proteins or control antigen (Linc-Bio Science Co. LTD, Shanghai, China), incubated overnight at 4°C, washed four times with PBST wash buffer (Tween-20 0.05% v/v in PBS), and blocked with 300 *μ*L of blocking buffer (bovine serum albumin 2% w/v in phosphate-buffered saline) for 2 h at 37°C. The plates were then washed four times with PBST wash buffer. 50-fold diluted serums (50 *μ*L) in blocking buffer were then added to antigen-coated wells and incubated for 1 h at 37°C. All the samples were tested in duplicate. The plates were thoroughly washed with PBST and then incubated with a 1 : 5000 dilution of anti-human IgG-horseradish peroxidase (Sigma, Poole, UK) at 37°C for 1 h. Then, the plates were thoroughly washed again four times, followed by the addition of 100 *μ*L of TMB substrate solution (0.04% TMB, 0.04% urea peroxide in 0.1 M sodium acetate-citric acid buffer, pH 4.0). After 10 min incubation in the dark at room temperature, the reaction was stopped by the addition of 50 *μ*L of 2 M H_2_SO_4_ to each well, and the values of the optical density at 450 nm (OD_450_) were measured with a microtiter plate reader (Bio-Rad, USA).

### 2.5. Statistical Analysis

The ELISA results were analyzed using cut-off values equal to the mean OD value for the healthy control serum samples plus three standard deviations (SDs). Any sample exhibiting absorbance above the cut-off value was considered to be positive. Sensitivity was determined by dividing the number of positive cases by the total number of TB patients. Specificity was determined by dividing the number of negative controls by the total number of healthy controls. For statistical analysis, the differences between different TB patients and healthy controls were calculated by the one-way ANOVA and independent-sample *t*-test using the Statistics Package for Social Science 17.0 (SPSS Inc., Chicago, IL, USA), with *P* < 0.05 considered to be significant. Furthermore, the receiver operation characteristic (ROC) curves of the OD values for antibody responses to each RD5-encoded recombinant proteins were plotted using the SPSS17.0 software; the areas of under the curve (AUC) were calculated, accordingly.

## 3. Results

### 3.1. Expression and Purification of RD5-Encoded Recombinant Proteins

**Figure 1:**
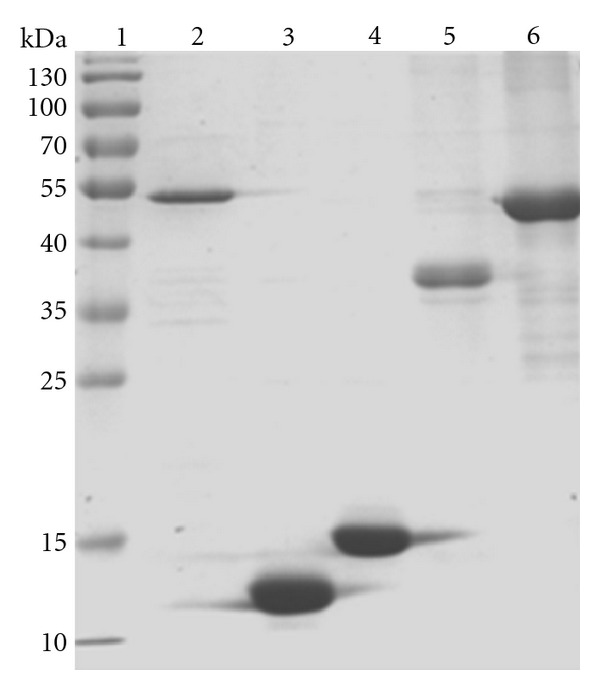
SDS-PAGE analysis of purified RD5-encoded recombinant proteins. Lane 1, molecular weight markers (SM0671, Fermentas, USA). Lane 2–6, purified Rv3117–Rv3121 recombinant proteins; molecular weight is 48.1 kDa, 11.2 kDa, 16.9 kDa, 38.2 kDa, and 45.3 kDa, respectively. Proteins were visualized with Coomassie brilliant blue staining.

To evaluate the antigenic ability of RD5-encoded recombinant proteins, the corresponding genes were expressed in *E. coli* BL21 (DE3) PLysS and purified as a His-tag fusion protein. The Rv3117, Rv3118, and Rv3119 recombinant proteins were largely present in the soluble fraction, and purification of them was carried out under non-denaturing conditions without urea. However, the Rv3120 and Rv3121 recombinant proteins were largely present in the insoluble fraction, and therefore the purification was carried out under denaturing conditions in the presence of 6 M urea, with a yield of 6.5 mg of protein/L of culture. The overexpressed N-terminal or C-terminal His-tagged recombinant proteins from RD5 genes were purified and confirmed by SDS-PAGE analysis (Figure 1). Then, they were dialyzed overnight against 20 mM Tris-HCl, pH 7.5, containing 100 mM NaCl and glycerol 3% (v/v) and used for immunoreactivity analyses.

### 3.2. Immunoreactivity Analyses of RD5-Encoded Recombinant Proteins

The serum IgG antibody responses to the RD5-encoded recombinant proteins or ESAT-6 antigen were compared with TB patients and healthy controls by ELISAs. Antibody responses to the individual protein in TB patients were analyzed with cut-off values obtained from 32 healthy individuals. As shown in Table 2, the sensitivity for detecting antibody responses to ESAT-6, Rv3117, Rv3118, Rv3119, Rv3120, and Rv3121 was 21.7%, 25.0%, 11.7%, 11.7%, 31.7%, and 5.0%, respectively, and the specificity was 90.6%, 96.9%, 90.6%, 96.9%, 96.9%, and 87.5%, respectively. Most importantly, the sensitivity and specificity of antibody detection by Rv3117 and Rv3120 were higher than those of antibody detection by ESAT-6 antigen. By combining the reactivity with recombinant proteins Rv3117 and Rv3120, a total test sensitivity of 35.0% for active TB patients was achieved, while good specificity (96.9%) still remained (Table 2).

**Figure 2:**
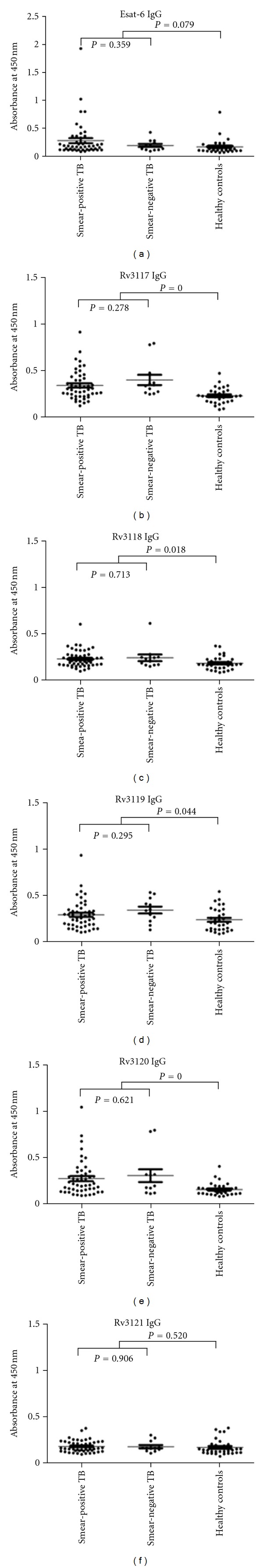
The humoral immune responses directed against the EAST-6 antigen (a) and Rv3117–Rv3121 recombinant proteins ((b)–(f)) were compared among different categories of patients and healthy controls. Horizontal lines indicate the means and the standard error of the mean of absorbent value each group. *P* values are shown above the plots determined by two-tailed one-way ANOVA and independent-samples *t*-test.

Furthermore, all the OD_450_ values of antibodies against RD5-encoded recombinant proteins and ESAT-6 antigen were used for the generation of ROC curves. The AUC of ESAT-6 antigen is 0.680 and those of Rv3117–Rv3121 recombinant proteins were 0.757, 0.656, 0.632, 0.735, and 0.609, respectively (figure not shown), suggesting Rv3117 and Rv3120 have a better overall diagnostic performance than any other individual antigen. In the study, all the TB patients compared with the healthy controls showed statistically significant responses against recombinant Rv3117 (*P* = 0.000), Rv3118 (*P* = 0.018), Rv3119 (*P* = 0.044), and Rv3120 proteins (*P* = 0.000) other than Rv3121 (*P* = 0.520) and ESAT-6 (*P* = 0.079). But antibody responses to EAST-6, Rv3117, Rv3118, Rv3119, Rv3120, and Rv3121 in the smear-positive TB patients were no stronger than that in the smear-negative TB patients (*P* > 0.05) (Figure 2). Meanwhile, the sera from smear-positive TB also showed a statistically significant response against ESAT-6 antigen (*P* = 0.047) compared with the healthy controls. However, the sera form the smear negative TB patients showed no statistically significant response against ESAT-6 antigen (*P* = 0.746) compared with the healthy controls (data not shown). Apparently, characterization of antibodies against recombinant proteins Rv3117 and Rv3120 can effectively distinguish between TB patients and healthy controls.

## 4. Discussion

The rapid screening test of active TB remains a serious problem in clinical practice, although it has been improved by the serodiagnostic methods [23]. These years, comparative genomic studies using subtractive DNA hybridization [19] and DNA microarray [20] have identified several *M. tuberculosis*-specific genomic regions of difference, designated RD1 to RD16, which are absent in the vaccine strains of *M. bovis* BCG, indicating that the RD-encoded recombinant proteins may be useful for improving the sensitivity and specificity of serodiagnosis TB. Accordingly, RD5 genes are present at *M. tuberculosis *but missed from BCG (BCG-Danish, BCG-pasteur, etc.) [20, 28]. Therefore, in this study, we successfully prepared five RD5-encoded recombinant proteins and evaluated by ELISA for their diagnostic potential in detecting serum antibodies comparison with ESAT-6 antigen which have been used for the diagnosis of TB [12, 14, 29] in a population of active TB patients and healthy controls.

Our results revealed that Rv3117 and Rv3120 were potential candidate antigens for the serodiagnosis of tuberculosis. The gene product of open reading frame Rv3117 of *M. tuberculosis* H_37_Rv was annotated as encoding a probable rhodanese-like thiosulfate sulfurtransferase, and it may be involved in intermediary metabolism and respiration [30–32]. And Rv3120-encoded protein was annotated as a conserved hypothetical protein. It was illustrated that the intactness of the RD5 region might be associated with increased virulence of the organism [33]. In this study, the sensitivity of detecting antibody responses to single recombinant protein Rv3117 or Rv3120 in TB patients and healthy controls was higher than that to ESAT-6 antigen, suggesting that they may be valuable for the rapid diagnosis of TB (Table 2). Notably, we found that no single serum sample reacted with six proteins tested and no single protein was recognized by all sera. These findings further indicated the heterogeneous antibody responses in tuberculosis [34] and the importance of the research on TB antibody profile for improving serodiagnosis of tuberculosis [35, 36], for any single *M. tuberculosis *antigen is not enough to be used to cover the antibody profiles of TB patients, but combinations of antigens may yield improved level of sensitivity, without affecting specificity. Since negligible anti-Rv3117 and anti-Rv3120 responses were obtained in the healthy controls; it was likely that they were expressed during the course of *M. tuberculosis* infection and that they may be associated with disease manifestation and progression. Our results also revealed that the specificity of Rv3117 and Rv3120 in healthy controls was higher than that of ESAT-6 antigen (Table 2). They both revealed a statistically significant antigenic distinction between healthy controls and TB patients (*P* < 0.001) (Figure 2). Therefore, the Rv3117 and Rv3120 recombinant proteins may be used for diagnosis of *M. tuberculosis* infection.

However, in a recent study, a total of 72 synthetic peptides covering the sequences of five RD5-encoded proteins had been tested by ELISAs for antibody reactivity with sera from HIV-negative pulmonary TB patients (*n* = 100) and *M. bovis* BCG-vaccinated healthy subjects (*n* = 100), but the pooled peptides were not strongly reactive with TB sera [37]. On the other hand, by using pools of overlapping synthetic peptides covering the sequence of RD5-encoded proteins, the CMI responses have been determined, the INF-*γ* data showed that the 11% responses in pulmonary tuberculosis patients and 2% responses in healthy blood donors, and the IL-10 data showed that the 33% responses in pulmonary tuberculosis patients and 17% responses in healthy blood donors, as discussed by Mustafa and Al-Attiyah [38]. Further studies are in progress to investigate the CMI response to recombinant protein Rv3117 and Rv3120, with the aim of evaluating its use in the cellular immunity-based immunodiagnosis of TB. And it is interesting to speculate on the possible use of the Rv3117 and Rv3120 proteins with other immunodominant antigens for vaccine development.

## 5. Conclusion

In this study, we successfully recombined five RD5-encoded proteins and evaluated for their diagnostic potential in detecting serum antibodies comparison with ESAT-6 antigen with sera from HIV-negative pulmonary TB patients and healthy control subjects. The results identified two immunodominant antigens, that is, Rv3117 and Rv3120, which show promise for use in the serodiagnosis of *M. tuberculosis*. They both revealed a statistically significant antigenic distinction between healthy controls and TB patients.

## Figures and Tables

**Table 1:** Primers and vectors used for cloning of RD5 genes.

Gene	Forward primer^a^ (5′–3′)	Reverse primer^a^ (5′–3′)	Restriction endonucleases used	Vectors	Annealing temperature (°C)	Amplicon size (bp)
Rv3117	CATACCATGGCACGCTGCGATG	CTTCTCGAGTCAGCTTCCCAAC	*Nco* I and *Xho* I	pET-32a	58	834
Rv3118	GGAATTCCATATGTGCTCTGGACCC	CTGCTCGAGGGTGATCTTGACGTCTAC	*Nde* I and *Xho* I	pET-21a	59	303
Rv3119	CATGCCATGGCCAATGTGGTAG	CTTCTCGAGTGGTCTATCGCCGAC	*Nco*1 and* Xho* I	pET-28b	57	444
Rv3120	CAAGGTACCATGAGTCCGTCTCCATCG	CCTAAGCTTCTACAGTGACCGTTGGGC	*Kpn* I and* Hind* III	pET-32a	63	603
Rv3121	GATATTCCATATGACAAGCACCTCG	CATAAGCTTCGTCGGCCAGGTAAC	*Nde* I and *Hind* III	pET-21a	58	1203

^a^The restriction sites used for cloning are underlined.

**Table 2:** Sensitivities and specificities for IgG antibodies against RD5-encoded *M. tuberculosis* proteins.

		Sensitivity			Sensitivity	
	Smear-positive TB (*n* = 48)	Smear-negative TB (*n* = 12)	All TB patients (*n* = 60)	BCG-vaccinated healthy control (*n* = 12)	PPD^−^ healthy control (*n* = 20)	All healthy control (*n* = 32)
ESAT-6^a^	25.0% (12/48)	8.4% (1/12)	21.7% (13/60)	83.4% (10/12)	95.0% (19/20)	90.6% (29/32)
Rv3117^a^	25.0% (12/48)	25.0% (3/12)	25.0% (15/60)	91.7% (11/12)	100.0% (20/20)	96.9% (31/32)
Rv3118^a^	12.5% (6/48)	8.4% (1/12)	11.7% (7/60)	91.7% (11/12)	90.0% (18/20)	90.6% (29/32)
Rv3119^a^	10.4% (5/48)	16.7% (2/12)	11.7% (7/60)	91.7% (11/12)	100% (20/20)	96.9% (31/32)
Rv3120^a^	29.2% (14/48)	41.7% (5/12)	31.7% (19/60)	91.7% (11/12)	100.0% (20/20)	96.9% (31/32)
Rv3121^a^	4.2% (2/48)	8.4% (1/12)	5.0% (3/60)	91.7% (11/12)	85.5% (17/20)	87.5% (28/32)
Rv3117 + Rv3120^b^	33.3% (16/48)	41.7% (5/12)	35.0% (21/60)	91.7% (11/12)	100.0% (20/20)	96.9% (31/32)

^a^The cut-off value was calculated from the mean OD value plus three standard deviations (SDs) for healthy controls.

^b^The serum tested was determined to be TB positive according to the criteria as follows: any antigen specifically reacts with serum when the cut-off value was calculated from the mean OD plus three standard deviations.
